# Evaluation of the Benefits and Risks of Introducing Ebola Community Care Centers, Sierra Leone

**DOI:** 10.3201/eid2103.141892

**Published:** 2015-03

**Authors:** Adam J. Kucharski, Anton Camacho, Francesco Checchi, Ron Waldman, Rebecca F. Grais, Jean-Clement Cabrol, Sylvie Briand, Marc Baguelin, Stefan Flasche, Sebastian Funk, W. John Edmunds

**Affiliations:** London School of Hygiene and Tropical Medicine, London, UK (A.J. Kucharski, A. Camacho, M. Baguelin, S. Flasche, S. Funk, W.J. Edmunds);; Save the Children, London (F. Checchi);; Milken Institute School of Public Health, George Washington University, Washington, DC, USA (R. Waldman);; Epicentre, Paris, France (R.F. Grais);; Médecins sans Frontières, Geneva, Switzerland (J.-C. Cabrol);; World Health Organization, Geneva (S. Briand)

**Keywords:** Ebola virus, Ebola outbreak, Western Area, Sierra Leone, western Africa, Ebola community care centers, transmission model, modeling, viruses, transmission, Ebola treatment centers, capacity, Ebola virus disease, epidemic

## Abstract

In some parts of western Africa, Ebola treatment centers (ETCs) have reached capacity. Unless capacity is rapidly scaled up, the chance to avoid a generalized Ebola epidemic will soon diminish. The World Health Organization and partners are considering additional Ebola patient care options, including community care centers (CCCs), small, lightly staffed units that could be used to isolate patients outside the home and get them into care sooner than otherwise possible. Using a transmission model, we evaluated the benefits and risks of introducing CCCs into Sierra Leone’s Western Area, where most ETCs are at capacity. We found that use of CCCs could lead to a decline in cases, even if virus transmission occurs between CCC patients and the community. However, to prevent CCC amplification of the epidemic, the risk of Ebola virus–negative persons being exposed to virus within CCCs would have to be offset by a reduction in community transmission resulting from CCC use.

## Abstract 2

These centers could lead to a decline in cases, even if virus containment is imperfect.

The current epidemic of Ebola virus disease in western Africa has resulted in thousands of cases during 2014 (*1*). To date, Ebola treatment centers (ETCs) have been used to isolate patients and provide clinical care. These facilities typically have large capacity (some have >100 beds) and function under high levels of infection control. However, in Sierra Leone, ETCs have reached capacity, and patients are being turned away (*1*). The reproduction number (defined as the average number of secondary cases generated by a typical infectious person) has been >1 in Sierra Leone, leading to growth in the number of cases reported each week (*2*–*4*). As a result, there is an urgent need to rapidly scale up treatment and isolation facilities. Delays in implementation will result in falling further behind the epidemic curve and in an even greater need for patient care facilities.

ETCs are complex facilities that require a substantial number of staff and time to set up; thus, the World Health Organization and other partners are looking at additional care options to supplement existing ETCs. One approach is the use of Ebola community care centers (CCCs), which would represent a possible change in operational approach (*5*–*7*). As envisioned in the World Health Organization approach, CCCs would be small units with 3–5 beds and would be staffed by a small group of health care workers. The main objective would be to isolate patients outside the home and, hence, reduce the movement and contacts of infectious persons within the community. CCCs are designed to engage the community and to increase the acceptance of isolation. Care for patients in CCCs would be provided primarily by a caregiver who would be given personal protective equipment (PPE) and basic patient care training. Patients would be free to leave the unit while awaiting test results. The specific utilization of CCCs would vary, depending on local context, and units would form part of a package of interventions, including monitoring of community contacts and burials within the community.

CCCs would be easier to set up than ETCs because they would be lightly staffed and could be made from local materials or even tents. Thus, CCCs have the potential to more rapidly begin treating patients. At present in Sierra Leone, the average time from symptom onset to hospitalization for Ebola virus disease patients is 4.6 days, which means patients remain in the community until the late stage of the disease (*4*). However, the use of CCCs has potential risks: the number of cases could be amplified if Ebola virus–negative patients in CCC assessment areas are exposed to infectious persons before admission, and virus could be transmitted between patients and caregivers or others in the community if virus containment within the CCC is not perfect. Given the urgent need for new operational solutions for Ebola patient care, it is critical to assess the conditions under which CCCs might exacerbate or mitigate the epidemic and to compare the scale-up of CCCs with the expansion of ETCs or home care.

We used an Ebola virus transmission model to evaluate the relative benefits and risks of introducing CCCs in a situation similar to that in Western Area, an administrative division of Sierra Leone. Western Area has exhibited consistent exponential growth in reported cases, and ETCs in the area are at capacity (*1*). Expert elicitation was used to estimate plausible values for key model parameters; these values were compared with simulation results to establish whether CCCs could be beneficial. We also estimated how many CCC beds, either alone or in combination with additional ETC beds, would be required to potentially turn over the epidemic (i.e., reduce the reproduction number below the critical threshold of 1).

## Methods

Because precise medical and operational details of CCCs are still under discussion, we focused on the implications of CCC introduction under a set of general assumptions. We modeled Ebola transmission by using a modified susceptible-exposed-infectious-resolved framework (*8*–*10*). In the model, persons were initially susceptible to the virus; upon infection, patients moved into a latent state for an average of 9.4 days (*4*) and then became symptomatic and infectious for an average of 9.5 days (*4*) before the disease was resolved (through either recovery or death and burial) and the patient no longer contributed to transmission. The model accounted for changes in ETC capacity to date (details available at https://drive.google.com/file/d/0B_BzCqSK1DZaYnRoeWtHOTU2TVk/). 

First, we used the model to generate epidemiologic forecasts for Western Area and to establish a baseline scenario for the level of infection if no additional interventions were introduced. We fitted the model to the number of weekly reported Ebola virus disease cases in Western Area during August 16–November 31, 2014 (*1*). We estimated that in Western Area the basic reproduction number (defined as the average number of secondary cases generated by a typical infectious person in the absence of control measures) was 1.94 (95% credible interval [CrI] 1.86–1.98) and that there would be 1,060 exposed persons (95% CrI 800–1,420) and 650 symptomatic persons (95% CrI 460–910) in the community on December 1, 2014.

**Figure 1:**
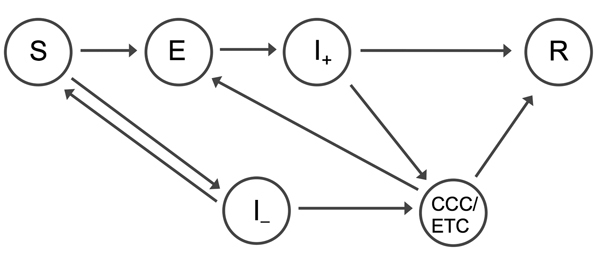
Structure of transmission model used to evaluate the benefits and risks of introducing CCCs into Western Area, Sierra Leone. Persons start off being susceptible to infection (S). Upon infection with Ebola virus, they enter an incubation period (E), and at symptom onset, they become infectious in the community (I_+_). After this point, infected persons seek health care in CCCs or ETCs; if centers are full, the infectious persons remain in the community until the infection is resolved (R) (i.e., the patients have recovered from the disease or are dead and buried). Patients admitted to ETCs and CCCs also move into the resolved compartment (R). We also assume that Ebola virus–susceptible persons could also become infected with other febrile diseases that have Ebola virus disease–like symptoms (I_–_). These Ebola virus–negative patients also seek health care; if centers are full, the patients return to the susceptible compartment (S) as symptoms wane. We assume the latent period is 9.4 days, the average time from symptom onset to CCC attendance is 3 days, and the average interval from symptom onset to ETC attendance is 4.6 days. CCCs, Ebola community care centers; ETCs, Ebola treatment centers.

To model the introduction of CCCs, we assumed that Ebola virus–susceptible persons could also become infected with other febrile diseases that have Ebola virus disease–like symptoms, which we assumed had symptoms that lasted an average of 7 days. Thus, 2 types of symptomatic persons were included in our simulation model: Ebola virus–positive and Ebola virus–negative patients (Figure 1; https://drive.google.com/file/d/0B_BzCqSK1DZaYnRoeWtHOTU2TVk/). In the model, Ebola virus–positive and –negative patients took an average of 4.6 days (*4*) after the onset of symptoms before attending an ETC. The probability that a patient was admitted to an ETC depended on the number of currently available beds. Well-managed ETCs operate strict patient isolation, careful use of PPE, and safe burial procedures (*11*,*12*), so we assumed that no virus transmission occurred between Ebola virus–infected patients and community members once patients were admitted to an ETC. If suspected case-patients were admitted to ETCs and subsequently found to be negative for Ebola virus, they returned to the community; we assumed there was no risk of Ebola virus–negative patients becoming infected while waiting for test results.

We also included CCCs in the model. We assumed that for patients visiting local CCCs, the time between symptom onset and CCC visit was shorter than that for patients visiting the larger and more distant ETCs; in the main analysis, we assumed that the average time from symptom onset to CCC attendance was 3 days. If CCCs were full, then patients attended ETCs instead. If ETCs were full, patients remained in the community. We assumed there was a possibility for some transmission of virus from CCC patients to community members (either directly, through caregivers, or during burial); we did not assume any transmission of virus from ETC patients. There was also a chance that Ebola virus–negative patients would be exposed to Ebola virus while waiting for test results. We assumed that 50% of symptomatic patients who attended CCCs/ETCs were Ebola virus–positive; on the basis of the number of Ebola virus disease cases and noncases reported in Sierra Leone, this percentage is plausible (*1*,*13*,*14*).

Two other parameters, besides the shorter time between onset of symptoms and attendance at a center, make CCCs potentially different from ETCs in the model: 1) the probability that Ebola virus–negative patients would be exposed to Ebola virus while waiting for test results in CCCs and 2) the reduction in virus transmission from infectious patients to the community as a result of the patient being isolated in a CCC. If the CCC model had a 100% reduction in transmission and 0% chance that Ebola virus–negative patients would be exposed virus, it was equivalent to the ETC set-up in the model, except that there would be a reduced time from symptom onset to CCC attendance.

## Results

**Figure 2:**
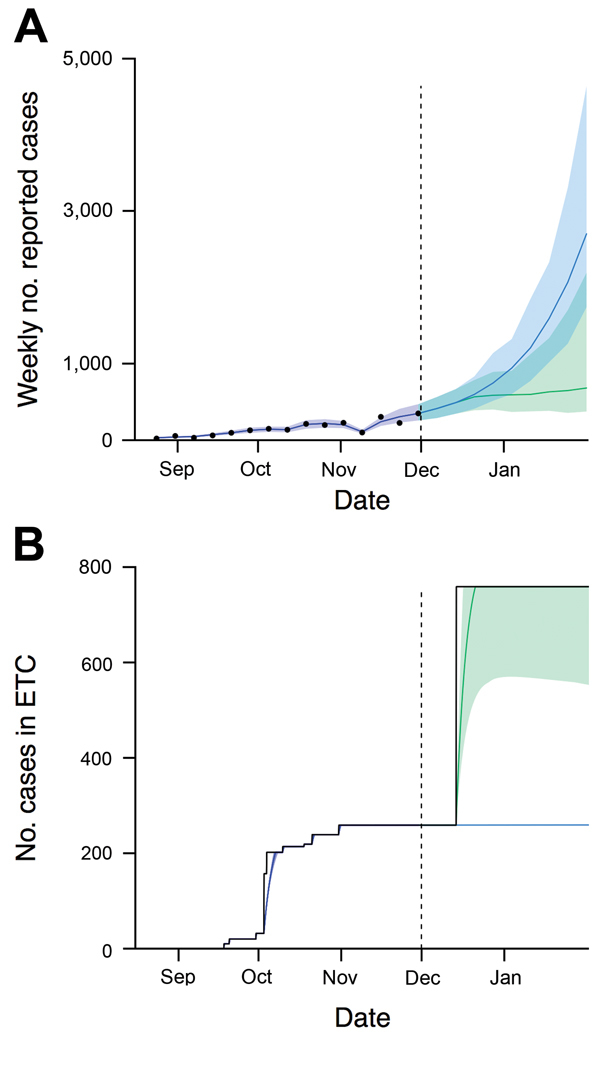
Model fits and forecasts used to evaluate the benefits and risks of introducing Ebola community care centers into Western Area, Sierra Leone. A) Reported cases over time. Black points show reported incidence data. B) No. patients in ETC beds. Blue lines to the left of the dashed vertical divides show the median estimate; blues line to the right of the dashed vertical divides show forecast with no change in number of ETC beds; green lines show forecast if 500 ETC beds are introduced on December 15, 2014. Shaded areas represent 95% credible interval, which reflects uncertainty about reporting and model parameters; darker shading indicates overlap between 2 forecasts. Estimates were scaled depending on the number of daily situation reports issued by the Sierra Leone Ministry of Health and Sanitation each week (see https://drive.google.com/file/d/0B_BzCqSK1DZaYnRoeWtHOTU2TVk/). ETC, Ebola treatment center.

We first considered the potential level of infection in the community during December 2014 based on our estimates for Western Area. With 259 ETC beds available (*1*,*14*–*16*), our model suggests that ETCs would be at capacity in mid-December and the number of cases would rise over the following weeks (Figure 2, panel A). We also considered the possibility that a proposed additional 500 ETC beds (*15*) would be introduced on December 15, 2014 (Figure 2, panel B). Our forecast suggested that the addition of these beds would cause the growth in number of cases to slow in the following weeks, but the change would not turn over the epidemic.

**Figure 3:**
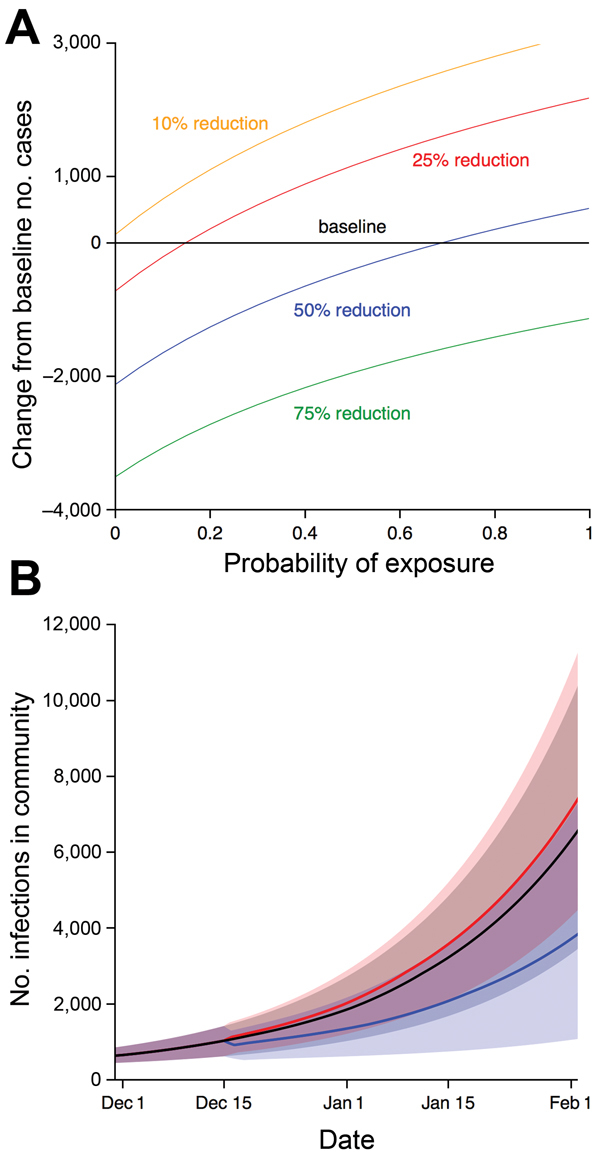
Factors influencing reduction or amplification of Ebola virus infection in the community if 500 CCC beds were introduced in Western Area, Sierra Leone, on December 15, 2014. A) Change in infection compared with baseline scenario (259 Ebola treatment center beds) between December 1, 2014, and February 1, 2015, for a range of values for reduction in transmission and probability of exposure to virus. Median parameter estimates for Western Area were used (Table). B) Change in infection over time. Black line, baseline scenario. Blue line, 500 CCC beds with transmission reduced by 75% (blue line in A), and Ebola virus–negative patients have 25% probability of exposure to virus. Red line, 500 CCC beds with transmission reduced by 25% (red line in A), and Ebola virus–negative patients have 50% probability of exposure to virus. Shaded areas show 95% bootstrapped credible intervals generated from 1,000 simulations with parameters sampled from posterior estimates. We assumed that time from symptom onset to CCC attendance was 3 days and that 50% of symptomatic patients were Ebola virus–positive. CCC, Ebola community care center.

To assess what reduction in transmission and in risk of Ebola virus–negative patient exposure to virus would be required for 500 CCC beds to be beneficial, we varied 2 key parameters and, after 30 days, compared model outputs with those for the baseline scenario (Figure 3, panel A). If there is a high probability that Ebola virus–negative patients will be exposed but only a small reduction in transmission, CCCs could act as incubators and generate more cases than the baseline scenario with 259 ETC beds only.

**Table:** Parameters used in a transmission model for evaluating the benefits and risks of introducing CCCs into Western Area, Sierra Leone*

Parameter	Value	Source
Mean time from symptom onset to outcome		
Ebola virus–positive patients	9.5 d	(*4*)
Ebola virus–negative patients	7.0 d	Assumed
Mean time from symptom onset to admission		
To ETC	4.6 d	(*4*)
To CCC	3.0 d	Assumed
Mean time from exposure to symptom onset (latent period)	9.4 d	(*4*)
Proportion of patients with Ebola-like symptoms in Western Area who are Ebola-positive	50.0%	(*1*)
Population of Western Area	1.4 million	(*17*)
Probability that an Ebola virus–negative patient seeking care in CCC will be exposed to Ebola virus	Varies†	NA
Reduction in transmission from infected patients to the community as a result of being in CCC	Varies†	NA
Basic reproduction no. (95% CrI)‡	1.94 (1.86–1.98)	Estimated
No. infectious persons on August 16, 2014 (95% CrI)§	51 (39.0–57.0)	Estimated
Proportion of cases in Western Area reported in Sierra Leone Ministry of Health situation reports (95% CrI)	0.42 (0.33–0.46)	Estimated
Variability in accuracy of reports, define as standard deviation of proportion of cases reported (95% CrI)	0.014 (0.010–0.024)	Estimated

*CCC, Ebola community care center; CrI, credible interval; ETC, Ebola treatment center; NA, not applicable. †In the analysis, the full range of possible values for these parameters is tested. ‡Basic reproduction number refers to the average number of secondary cases generated by a typical infectious patient at the start of an epidemic §This parameter represents the initial no. of infectious patients at the start of the model simulation. Additional information is available at https://drive.google.com/file/d/0B_BzCqSK1DZaYnRoeWtHOTU2TVk/.

The CCC approach has not been fully tested in the field, so we conducted an elicitation of 6 expert opinions to obtain estimates for the median and interquartile range (IQR) for reduction in transmission as a result of patients being in CCCs and for the probability of exposing Ebola virus–negative patients to infectious patients (details at https://drive.google.com/file/d/0B_BzCqSK1DZaYnRoeWtHOTU2TVk/). The distribution for the group opinion for reduction in transmission while in a CCC had a median of 63% (IQR 41%–81%). The distribution for the probability of exposure had a median of 0.09 (IQR 0.01–0.36). When compared with model results, these estimates were within the region of parameter space in which CCCs would be beneficial (see Figure 3 at https://drive.google.com/file/d/0B_BzCqSK1DZaYnRoeWtHOTU2TVk/).

To confirm that 63% was a plausible value for reduction in transmission, we used the following theoretical argument. In the model, the basic reproduction number, *R*_0_, was near 2, the time from onset to outcome was 9.5 days on average, and patients took an average of 3 days after onset of symptoms to attend CCCs. If infected persons did not enter an available CCC and instead remained in the community for the next 6.5 days, they would generate an average of 1.4 secondary cases (because 2 × 6.5/9.5 = 1.4). Even if Ebola patients had a 50% probability of infecting their sole caregiver, it meant they would, on average, generate 0.5 secondary cases while in a CCC. The relative reduction in cases as a result of being in a CCC would therefore be (1.4 − 0.5)/1.4 = 64%. If each case-patient generated an average of 0.25 cases while in a CCC, the expected reduction would be ≈80%.

To elucidate the potential benefits and risks of CCC introduction, we considered 2 specific examples. If CCCs reduced virus transmission from Ebola virus–infected patients to the community by 75% once the patient was admitted and if Ebola virus–negative patients have a 25% probability of exposure while waiting for test results, then the introduction of 500 CCC beds would slow virus transmission (Figure 3, panel B). However, if CCCs only reduced transmission by 25% and Ebola virus–negative patients have a 50% probability of exposure to Ebola virus, the introduction of 500 CCC beds could lead to a rise in the number of cases within the community (Figure 3, panel B).

**Figure 4:**
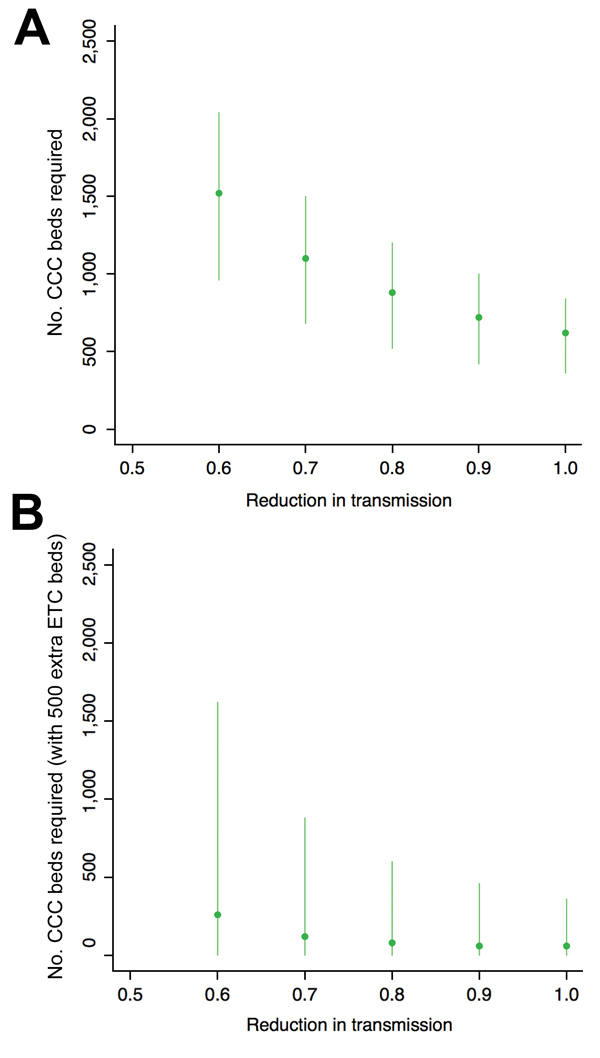
Estimated number of CCC beds required to control Ebola virus epidemic in Western Area, Sierra Leone. A) Number of CCC beds required to turn over the outbreak (i.e., reduce the reproduction number, *R*, to <1). When transmission is reduced by only 50%, no amount of CCC beds can stop the growth in cases. We assume there is a 10% probability that Ebola virus–negative patients are exposed to virus. Lines show bootstrapped 95% credible intervals generated from 1,000 simulations with parameters sampled from posterior estimates; points show median estimates. B) Number of CCC beds required to turn over the epidemic when an additional 500 Ebola treatment center beds are also introduced on December 15, 2014. CCC, Ebola community care center.

We also assessed how many CCC beds would be required to stop the exponential increase in cases and turn over the epidemic (i.e., reduce the reproduction number of the infection, *R*, to <1). A larger number of beds would be required if the reduction in transmission was smaller (Figure 4, panel A). The requirement was also larger if Ebola virus–negative patients were more likely to be exposed to virus, patients took longer to attend CCCs, or there were more Ebola virus–negative patients (see figures 4 and 5 at https://drive.google.com/file/d/0B_BzCqSK1DZaYnRoeWtHOTU2TVk/). The large number of infected persons on December 1, 2014, meant that the number of cases still rose in the model (Figure 2), suggesting additional interventions would be required to control the epidemic. Therefore we assessed a combination of the 2 health care approaches, with additional ETC beds, CCC beds, or both introduced on December 15, 2014 (Figure 4, panel B). Because CCCs reduce the time from symptom onset to attendance at a health care center, our results suggest it would be possible to turn over the epidemic in Western Area with a sufficient number of CCC beds, either as a standalone strategy or in combination with additional ETCs.

## Discussion

We used a transmission model to evaluate the potential effects of the introduction of Ebola CCCs in Western Area, Sierra Leone. Our results show that CCCs could reduce the number of Ebola virus disease cases in the community if 1) the probability for Ebola virus–negative patients being exposed to the virus is low and 2) there is reduction in virus transmission as a result of infected patients being in CCCs. The introduction of CCCs could potentially turn over the epidemic (i.e., reduce the reproduction number, *R*, below the critical threshold of 1) if the time from symptom onset to CCC attendance is <3 days. Assuming that CCCs open in mid-December, ensuring epidemic turnover would require a large number of CCC beds (potentially at least 500 for Western Area). In addition to reducing the time from symptom onset to attendance at a treatment facility, a large number of CCCs would have the added benefit of reducing the time from symptom onset to admission because infected patients would not have to wait for ETC beds to become available.

Our analysis does have limitations. One of those limitations is that we used an illustrative scenario for Western Area based on current epidemiologic reports. Given uncertainty about the influence of factors such as changes in behavior (*18*), we focused our analysis on short-term forecasts and estimation of the number of beds required to turn over the epidemic. However, the epidemiologic landscape is changing rapidly, and the situation might have been different by late December/January, which would influence our specific estimates for bed requirements. In addition, transmission dynamics may vary by district, which would influence the precise number of beds required in different areas. Our results should therefore be viewed as qualitative rather than quantitative. In addition, the reduction in transmission as a result of patients being in CCCs will, in reality, depend on several factors, including patient movements, PPE effectiveness, infection control in the facility, and burial procedures (*12*), and these factors will likely differ between settings. Because it was not possible to establish the contribution of each factor to disease transmission without detailed data on the source of infection (*8*), we used a single parameter to capture the reduction in transmission as a result of a patient being in a CCC. Given the uncertainty about the precise magnitude of this reduction, we assessed the effect of CCCs under the full range of potential reductions in transmission, from no change to full containment, and conducted an elicitation of expert opinions to identify plausible parameter ranges.

Furthermore, we assumed that infectiousness does not vary over the course of Ebola virus infection. However, if patients are most infectiousness during the final stages of infection (*19*,*20*), then CCCs and ETCs would provide an even greater reduction in transmission because they would isolate patients when they are most infectious. In addition, it has been shown that it is not possible to reliably estimate multiple routes of transmission for Ebola virus from a single incidence curve (*8*); thus, we chose to model community transmission by using a single parameter, rather than attempting to estimate the contribution from living infected persons and from funerals. In the model, we also assumed that all patients seek health care. If in reality some do not, this will have the effect of increasing the average time from symptom onset to admission in a care center. A crucial point is that if patients on average spent more than half of their infectious periods in the community, then expansion of bed capacity alone would not be enough to turn over the epidemic in regions where the reproduction number is near 2.

In summary, CCCs may offer a rapid, high-coverage complement to ETCs and, thus, hold considerable potential for bringing about a sizeable shift in the epidemic pattern in Sierra Leone. The UK government is therefore supporting such a combined intervention in the Sierra Leone (*7*). However, the CCC approach is little tested in the field and could be harmful if infection control in CCCs is worse than that in the community or if Ebola virus–negative patients have a high risk of exposure to virus. Settings with limited triage, such as primary health care facilities, may also expose Ebola virus–negative patients to the virus and could therefore also have the potential to amplify the Ebola epidemic. Given the potential benefits and risks of introducing CCCs, real-time evaluation of their effectiveness must be carried out as they are implemented. In particular, to confirm the usefulness of CCCs as an epidemic control strategy, estimates must be determined for the reduction in virus transmission as a result of infected patients being isolated in CCCs and for the probability of Ebola virus–negative patients being exposed to virus in CCCs.
